# Non-convulsive Status Epilepticus as a Complication of Electroconvulsive Therapy: A Case Report and Review of the Literature

**DOI:** 10.7759/cureus.35639

**Published:** 2023-03-01

**Authors:** Tuğçe Saltoğlu, Bedirhan Şenol, Guray Koc

**Affiliations:** 1 Neurology, Ankara City Hospital, Ankara, TUR; 2 Psychiatry, Ankara City Hospital, Ankara, TUR

**Keywords:** clozapine, schizophrenia, epilepsy, non-convulsive status epilepticus, electroconvulsive therapy

## Abstract

Electroconvulsive therapy (ECT) is a highly effective treatment option among the biological treatments introduced in psychiatry. It has been used successfully to treat neurological conditions such as epilepsy, Parkinson's disease, and major psychiatric disorders. Non-convulsive status epilepticus can be seen as a complication after ECT, although it rarely occurs. Due to its rarity, this complication is not yet well understood, is challenging to diagnose, and information about treatment options is limited. Here, we present a 29-year-old patient with no previous neurological disease and a history of schizophrenia and refractory psychosis on clozapine; nonconvulsive status epilepticus was detected in the EEG after ECT.

## Introduction

Non-convulsive status epilepticus (NCSE): general overview

Status epilepticus (SE) is defined as a condition that can have long-term outcomes involving neuronal death and injury depending on the variety and continuance of seizures due to the failure of the mechanisms responsible for seizure termination or from initiating mechanisms that lead to abnormally prolonged seizures [1]. According to the International League Against Epilepsy (ILAE), SE without obvious motor symptoms is delineated as NCSE and it is primarily sorted out according to the level of consciousness (NCSE with coma and without coma). NCSE without coma is also classified as generalized, focal, or indeterminate [1,2].

Salzburg consensus criteria for diagnosis of NCSE in patients without known epileptic encephalopathy recommends demonstrating specific EEG features, subtle clinical phenomena, and clinical and EEG improvement after antiepileptic drug (AED) use [3,4].

NCSE usually demonstrates the deterioration of consciousness ranging from mild confusion to coma and less commonly exists as focal SE without impairment in consciousness. In addition, other clinical manifestations are categorized as positive and negative symptoms: negative symptoms, which appear such as anorexia, aphasia, amnesia, and catatonia, and positive manifestations such as agitation, aggression, nystagmus, perseveration, psychosis, or tremulousness [5].

Medical history is a critical part of the diagnosis in NCSE patients. Particularly, previous substance abuse, psychiatric illness, head trauma, infection, and use of antipsychotics that lower the seizure threshold such as clozapine, stroke, meningitis, encephalitis, brain tumor, neurosurgery, and dementia are linked to increased risk [6].

There is no specific treatment guideline for NCSE yet, and it is a matter of debate whether it can be treated as aggressively as convulsive SE [7]. Initial treatment in patients with NCSE is a combination of an antiepileptic drug (valproic acid, fosphenytoin, lacosamide, and levetiracetam) that will not induce coma and a benzodiazepine. NCSE treatment should be monitored with continuous EEG under close clinical supervision [8].

NCSE as a complication of electroconvulsive therapy (ECT)

ECT is a well-tolerated treatment option that has been in practice for 80 years and usually has mild side effects. It is mainly used in psychiatric conditions such as mania, depression with suicidal tendencies, and a catatonic or medication-refractory state. Side effects related to ECT usually occur shortly after the session and may be due to the anesthetic agent, anticholinergic drug, muscle relaxant, or electrical stimulation applied during the procedure. Side effects are grouped within themselves; general side effects such as headache and nausea and side effects due to the applied muscle relaxant such as seizure-related and cardiovascular side effects. Seizure-related side effects may be prolonged or tardive seizures. Prolonged or tardive seizures may, in rare cases, convert into convulsive SE or NCSE. NCSE may be demonstrated as varying degrees of abnormalities in mental state or conduct after ECT and must be distinguished from postictal confusion [9].

## Case presentation

A 29-year-old male patient with schizophrenia had a 12-packs per year smoking history. The patient who previously got along well with his family but had limited social participation started thinking that his friends and teachers at school were talking about him; he heard them insult him after he used cannabis with his roommates at university about five years ago. In the following years, he became a more introverted person and started to remain confined to the house. In this way, his first psychiatric complaints were auditory-type hallucinations and persecution. The patient, who was hospitalized for exacerbations of psychosis with the same symptoms such as auditory hallucinations and persecution in each episode four times in five years, had a history of ECT because of his excitations, and drug side effects in the third year of his illness. In the following two years, while he was preparing for a vocational exam, his symptoms reappeared as thoughts that someone was after him, hearing negative thoughts from his upstairs neighbors about him, and insomnia. The amount of smoking increased from an average of one pack per day to three to four packs per day. He would be disturbed by television and telephones and open the door frequently to see if anyone was after him. He presented to our psychiatry clinic and was hospitalized.

In the mental state examination of the patient, it was observed that he was looking old, his self-care was decreased, he was irritable, there were auditory hallucinations in perception, he had reality distortion, there were themes consistent with persecution, and referential delusions in his thoughts. His positive and negative syndrome scale (PANSS) score was calculated as 106 at his admission. Patient history was not clear why the clozapine dose continued at 225 mg/day during the follow-ups for treatment planning and the dose was not increased; amisulpride 200 mg/day was added to his treatment, and it was slowly increased to 800 mg/day. No abnormality was detected in the magnetic resonance imaging of the brain. After two weeks, auditory hallucinations and persecutory delusions did not subside, and the visiting team decided to slowly increase the dose of clozapine. While he was receiving clozapine 400 mg/day and amisulpride 800 mg/day treatment, he had risky behaviors such as putting out cigarettes on himself and threatening other patients, and his complaints did not regress, so his smoking was limited to one pack per day for a temporary period and it was decided to apply ECT treatment, which he had previously benefited from. Consent was obtained from him and his family. After the sixth session of ECT treatment, the PANSS score was evaluated as 69. As he benefited from ECT treatment but his complaints did not completely regress, it was decided to complete the ECT treatment in 10 sessions as in his previous hospitalization. 

**Figure 1:**
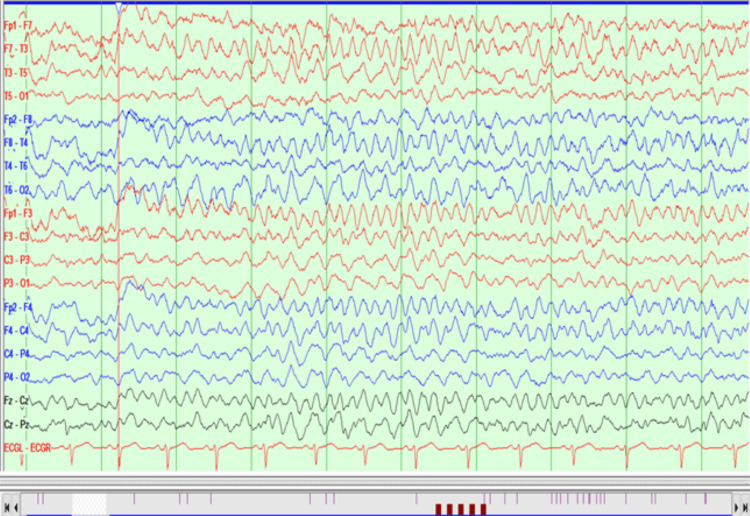
First EEG (sensitivity is 7 μV/mm) of the patient; right temporal rhythmic delta activity spreads to the left homolog area three seconds later, and the amplitude increases. The duration of this activity lasted 15 seconds.

EEG was planned for the patient with no dissociative or depressive disorder found in the past due to urinary incontinence and persisting hallucinations and psychotic symptoms after the ninth session of ECT. Generalized slow wave activity and intermittent rhythmic delta activity at a frequency of 2.5-3 Hz, sometimes lasting longer than 10 seconds, were observed in the EEG; therefore, it was found suspicious for NCSE (Figure 1) and a second EEG was planned by administering diazepam to confirm the diagnosis. 

**Figure 2:**
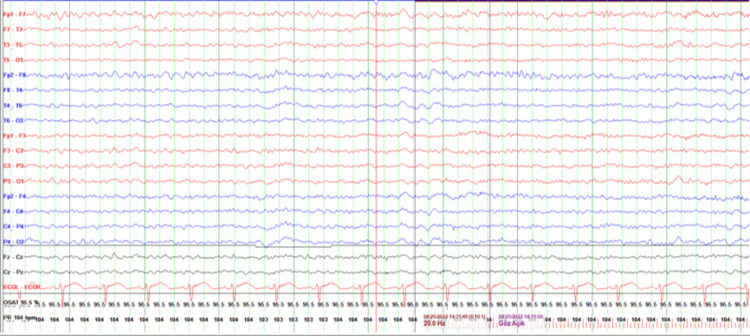
In the EEG (sensitivity is 7 μV/mm) activity under the treatment of midazolam, no abnormal activity was seen.

**Figure 3:**
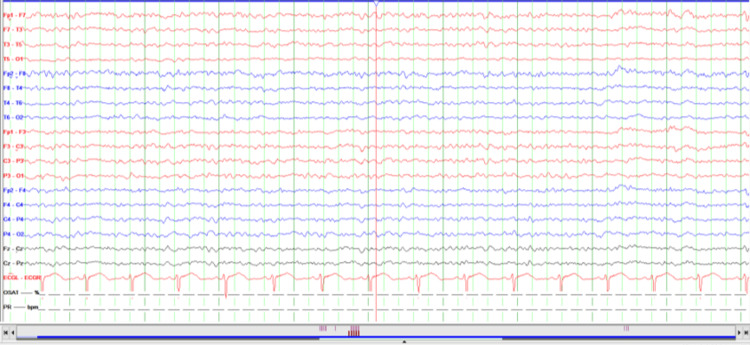
Last EEG (sensitivity is 7 μV/mm) before the discharge.

During the second EEG, the patient's visual hallucination consistent with delirium including flower picking was detected. After diazepam, the patient whose EEG tracing was improved (Figures 2, 3), was admitted to the neurology intensive care unit with a preliminary diagnosis of NCSE. He was followed up for 48 hours with continuous 4 mg/hour/day midazolam and continuous bedside EEG in the neurology intensive care unit. Concomitant lamotrigine was started at 100 mg/day. Significant improvement in EEG, sinusoidal alpha, and beta waves with eyes open was observed at the 48th hour, and the patient was transferred to the psychiatry service.

Lamotrigine treatment was increased up to 200 mg/day and clozapine 350 mg/day was adjusted in the psychiatry service after he was taken over from the neurology intensive care unit. Considering the risk of side effects, clozapine blood level was requested from the patient and the results was clozapine 241.8 ng/mL (the defined therapeutic range is 350-600 ng/mL). The PANSS score was evaluated as 57 at the time of discharge.

## Discussion

The diagnosis of NCSE post-ECT can be laborious; the symptoms may not be characteristic and clear, and are usually not distinguishable from symptoms of confusion, delirium, or psychiatric illness; hence, the follow-up psychiatrist should be careful. In suspicious cases, EEG should be performed, especially in patients at risk for seizures. These risky conditions include previous seizure history, and lithium or clozapine use [9]. Our patient's psychotic symptoms that are resistant to ECT support the diagnosis of NCSE. In addition, the hallucination of picking flowers noticed is not a typical hallucination for schizophrenia, but rather supports delirium, a positive symptom of the NCSE; on the other hand, EEG changes occurring also distinguish the clinic from dissociative hallucinations [4,10]. Complex partial seizures of temporal origin could be presented with psychopathology also and this was in our differential diagnosis; however, aura and automatism were not observed in our case [11]. 

These patient symptoms were compatible with type 3b according to the modified Salzburg consensus criteria for NCSE, and the improvement seen in the patient's EEG with rhythmic activity after IV AEDs also confirms the diagnosis [3].

It is known that clozapine is linked to an increased risk of seizure depending on dosing, serum concentration, and pharmacokinetic/pharmacodynamic interactions. Seizure risk is reported to occur in association with clozapine, with a cumulative one-year risk of approximately 5% [12]. Clozapine is an antipsychotic that is effective on positive and negative symptoms of schizophrenia and induces CYP1A2 and CYP3A4 enzymes. The most important side effects are interaction with many drugs and substances due to metabolism, agranulocytosis, and neutropenia. It is known that smoking increases clozapine levels and is prone to drug interactions. Our patient was using clozapine 400 mg/day and was a chronic smoker. Still, his smoking was limited due to putting out the cigarette on his body while internalizing in the hospital. This may have lowered the seizure threshold by increasing the blood level of clozapine. Seizures may occur when the clozapine blood level is above 500 μg/l and severe seizures may occur above 1300 μg/l. Although our patient's final clozapine value was low, 241.8 ng/mL, we do not know the clozapine value at the time of the patient's diagnosis of NCSE [13]. In addition to all these, the increased risk of seizures may also be associated with the type of ECT. Studies and case reports have shown that bi-frontal ECT can be associated with a higher increased risk of seizures than bi-temporal ECT [9,14]. Bi-frontal ECT was given to our patient.

As treatment for our patient, we started midazolam infusion and lamotrigine 100 mg/day peroral in the acute period. We preferred lamotrigine because it has mood-stabilizing and antidepressant properties, and in various case reports, lamotrigine was used in seizures caused by clozapine. We did not prefer valproic acid (Class IIb, level A) with clozapine because of its potential to trigger agranulocytosis. There are cases reporting that the risk of agranulocytosis and neutropenia clearly increased with valproate used in combination with clozapine. However, we did not prefer valproic acid for levetiracetam (Class IIb, level C) in terms of its psychiatric side effects [3-13].

NCSE after ECT is a rare and not yet clarified condition. We retrieved a review article by Aftab et al. in which cases between 1980-2005 were examined [9]. In this review, they identified 13 cases and EEG findings were detected to support the diagnoses of all. Clinical presentation was usually altered mental status or unresponsiveness and also all cases were detected to have risk factors such as prolonged seizures, convulsive SE, and use of antipsychotics and antidepressants; it is noteworthy that most of the cases were female. It was observed that a combination of iv benzodiazepine and antiepileptic, which did not induce sedation, was used, as in the treatments we applied to our patient.

We reviewed English-language MEDLINE/PubMed articles containing the keywords ‘‘nonconvulsive status epilepticus’’ and ‘‘electroconvulsive therapy’’. Articles including NCSE patients post-ECT were included since the year 2005. We retrieved two more case series. The first was by Weis et al. At age 48, one of their patient had post-ECT delirium, was disoriented with fluctuating attention, intermittent hand, and verbal automatisms, and was notable for absent paranoid delusions. In this patient, bi-temporal ECT was administered and the patient was using clozapine [15]. In the second case series by Reyes-Molón et al., an 83-year-old female patient had electrical SE in the third session of ECT; temporal or frontal leads of ECT were not specified [16]. One of the most exceptional details was seen in a case series by Povlsen et al., in which peroral clonazepam was administered for the treatment of NCSE [17], as opposed to our observation that clozapine lowered the seizure threshold in some of the cases, including our case. Table 1 gives a comparison of case summaries.

**Table 1:** Update of case summaries

Reference	Diagnosis	Age, sex	Clinical features	Treatment
Weiss et al. [15]	Schizoaffective disorder	48 years, Female	Delirium, disorientation with fluctuating attention, intermittent hand, and verbal automatisms	Sodium valproate and topiramate
Reyes-Molón et al. [16]	Psychotic depression	83years, Female	Only EEG findings	Valium,penthotal and propofol
Pogarell et al. [17]	Depression	55 years, Female	Apathy, confusion, disorientation, amnesia	Lorazepam
Povlsen et al. [18]	Bipolar Disorder	29 years, Female	Confusion,disoriantation	Phenytoin, diazepam,oxcarbazepine
Povlsen et al [18]	Depression	28 years, Female	Aggression, memory loss, confusion disorientation, myocloni in extremities	Diazepam,oxcarbazepine,clonazepam
Povlsen et al [18]	Depression	29 years, Female	Confusion, disorientation, apraxia	Diazepam,oxcarbazepine,clonazepam
Smith and Keepers [19]	Depression	87 years, Female	Confusion, slight clonic movements of the face	Lorazepam, phenytoin
Srizch and Turbott [20]	Depression	40 years, Female	Confusion, mental retardation, disorientation, slow speech	Diazepam, phenytoin, carbamazepine
Solomons et al. [21]	Depression, panic disorder, agoraphobia	82 years, Female	Psychomotor retardation	Diazepam, phenytoin
Grogan et al. [22]	Depression with psychotic features	34 years, Female	Confusion, fasial paresis	Diazepam, phenytoin,valproate
Dean et al. [23]	Depression, anxiety	76 years, Male	Unresponsiveness	Diazepam, midazolam, thiopental, phenytoin
Hansen Grant et al [24]	Depression with psychotic features	70 years, Male	Unresponsiveness to pain, perioral twitching, and some systemic features	Midazolam, phenytoin
Hansen-Grant et al. [24]	Psychosis, intellectual disability	18 years, Male	Unresponsiveness to pain, eye-rolling movements, dilated pupils	Diazepam, mannitol,phenytoin
Hansen-Grant et al. [24]	Depression with psychotic features	65 years, Female	Apathy hemiparesis, lip-smacking, jerking	Diazepam, phenytoin, valproate
Hansen-Grant et al. [24]	Depression with psychotic features	33 years, Female	Confusion, disorientation, unresponsiveness	Diazepam, valproate
Current case	Schizophrenia	29 years, Male	Confusion, delirium, urinary incontinence	Midazolam, lamotrigine

## Conclusions

The diagnosis of NCSE after ECT is a demanding/critical/obscure condition. Particular attention should be paid to factors that will lower the seizure threshold like treatment with clozapine. In cases with ECT treatment with clozapine, intermittent clozapine blood levels can be quantified and medication interactions and smoking should be considered. It is important to recognize NCSE after ECT since the results are good when treated if the diagnosis was made properly.
